# Factors affecting postural instability after more than one-year bilateral subthalamic stimulation in Parkinson’s disease: A cross-sectional study

**DOI:** 10.1371/journal.pone.0264114

**Published:** 2022-02-23

**Authors:** Andrea Kelemen, László Halász, Loránd Erőss, Gábor Rudas, Muthuraman Muthuraman, Dénes Zádori, Bence Laczó, Dávid Kis, Péter Klivényi, Gábor Fekete, László Bognár, Dániel Bereczki, Gertrúd Tamás

**Affiliations:** 1 Department of Neurology, Semmelweis University, Budapest, Hungary; 2 National Institute of Clinical Neurosciences, Budapest, Hungary; 3 MR Research Centre, Semmelweis University, Budapest, Hungary; 4 Biomedical Statistics and Multimodal Signal Processing Unit, Department of Neurology, University Medical Center of Johannes Gutenberg University Mainz, Mainz, Germany; 5 Department of Neurology, University of Szeged, Szeged, Hungary; 6 Department of Neurosurgery, University of Szeged, Szeged, Hungary; 7 Department of Neurosurgery, University of Debrecen, Debrecen, Hungary; Universidade de Sao Paulo Faculdade de Medicina, BRAZIL

## Abstract

**Background:**

Balance impairment in Parkinson’s disease is multifactorial and its changes due to subthalamic stimulation vary in different studies.

**Objective:**

We aimed to analyze the combination of predictive clinical factors of balance impairment in patients with Parkinson’s disease treated with bilateral subthalamic stimulation for at least one year.

**Methods:**

We recruited 24 patients with Parkinson’s disease treated with bilateral subthalamic stimulation and 24 healthy controls. They wore an Opal monitor (APDM Inc.) consisting of three-dimensional gyroscopes and accelerometers in the lumbar region. We investigated four stimulation conditions (bilateral stimulation OFF, bilateral stimulation ON, and unilateral right- and left-sided stimulation ON) with four tests: stance on a plain ground with eyes open and closed, stance on a foam platform with eyes open and closed. Age, disease duration, the time elapsed after implantation, levodopa, and stimulation responsiveness were analyzed. The distance of stimulation location from the subthalamic motor center was calculated individually in each plane of the three dimensions. We analyzed the sway values in the four stimulation conditions in the patient group and compared them with the control values. We explored factor combinations (with age as confounder) in the patient group predictive for imbalance with cluster analysis and a machine‐learning‐based multiple regression method.

**Results:**

Sway combined from the four tasks did not differ in the patients and controls on a group level. The combination of the disease duration, the preoperative levodopa responsiveness, and the stimulation responsiveness predicted individual stimulation-induced static imbalance. The more affected patients had more severe motor symptoms; primarily, the proprioceptive followed by visual sensory feedback loss provoked imbalance in them when switching on the stimulation.

**Conclusions:**

The duration of the disease, the severity of motor symptoms, the levodopa responsiveness, and additional sensory deficits should be carefully considered during preoperative evaluation to predict subthalamic stimulation-induced imbalance in Parkinson’s disease.

## Introduction

The long-term effects of subthalamic stimulation (STN DBS) on postural instability in Parkinson’s disease (PD) still need exploration.

In PD, different elements of balance control (balance during quiet stance, the reactive postural adjustments to external perturbations, the anticipatory postural adjustment in preparation for voluntary movements, and the dynamic balance during movements) are abnormal, and their interaction disposes patients towards falls [1]. Balance impairments were documented already in the prodromal [2] and the early stage of Parkinson’s disease [3] and increased with disease progression [4], along with the neurodegeneration in dopaminergic and non-dopaminergic networks [5,6]. They contribute to disability [7] and falls [8]. Integration disturbances of vestibular [9], proprioceptive and visual information, altered background muscle tone covering stooped posture [10], abnormal patterns of motor adjustment, orthostatic hypotension, frontal executive dysfunction, and even mild cognitive impairment [11] may interfere with reconciliation of normal balance mechanisms in PD [1,12]. Furthermore, static imbalance increases with age, even in healthy subjects with closed eyes [13]. Additionally, an altered step strategy, biomechanical impairments [14], and possible comorbidities play a role in imbalance with older age [15]. Levodopa therapy further worsens balance as the disease progresses [5,16,17]. Subthalamic stimulation has a beneficial effect on postural instability in the first 9–12 months of the therapy, which wanes over time [18–21] as a result of a possible neuromodulatory effect [21] or as a result of the disease progression [18]. Nevertheless, cross-sectional studies reported a positive effect of STN DBS on axial symptoms, even 6–18 months [22], 3–69 months [23], and 36±21.6 months [24] after surgery in patient cohorts. It was also observed that switching the STN DBS on may improve or worsen balance in individual patients [25]. Its underlying causes are still unclear as earlier studies assessed different elements of the balance control with diverse sample sizes and methods (S1 Table).

Several studies have analyzed the influence of STN DBS on balance within the first year of the postoperative phase [16,25–30]; therefore, the early benefits and not the long-term effects have been investigated. Additionally, only a few studies on balance have analyzed the influence of the active contact location [18,25,31]. Multiple clinical factors and their possible combinations contributing to changes in postural instability have not yet been investigated. Its mechanism has also not been explored.

In the present study, we hypothesized that factor combinations from disease-related variables and individual location of the active contacts are predictive for developing static imbalance while switching the stimulation on in patients with long-term STN DBS therapy. We used motion sensors to describe stance in quiet and proprioceptive, visual sensory conflict situations quantitatively to investigate if visual or proprioceptive information dependency is more characteristic of stimulation-induced imbalance.

## Materials and methods

### Participants

We recruited 24 PD patients treated with bilateral STN DBS and an age-matched group of 24 healthy controls. Exclusion criteria were significant orthopedical/rheumatological disorders or visual disability not correctable with eyeglasses.

The Core Assessment Program for Surgical Interventional Therapies for Parkinson’s Disease [32] was followed when indicating the surgery. The inclusion criteria of the PD patients with DBS treatment were as follows: there were at least 12 months elapsed since the operation, stable stimulation parameters and clinical state for at least 3 months.

For individual anatomical planning, preoperative contrast-enchanced MR (3T Phlilips Achieva) images and stereotactic contrast-enchanced CT sequences (made on the day of surgery) were merged using the Medtronic FrameLink 5 software. Intraoperative electrophysiological mapping was executed with five microelectrodes. Clinical symptoms were controlled through macrostimulation [33].

Ethical approval (reference number: 271/2013) was obtained from the Regional and Institutional Committee of Science and Research Ethics, Semmelweis University and patients signed informed consent forms.

### Measurement protocol

We used a wireless Opal monitor (APDM Inc.) consisting of three-dimensional gyroscopes and accelerometers placed on the lumbar region to measure sway [34]. The sample rate was 128Hz.

The patients executed the Instrumental Clinical Test of Sensory Integration and Balance (ICTSIB), each part of which lasted for 3x30 seconds: stance on a plain ground with arms folded across the chest with eyes open (OG) and eyes closed (CG), stance on foam with arms folded across the chest with eyes open (OF) and eyes closed (CF) in randomized order. Feet position was set with a foot block in every patient.

**Fig 1:**
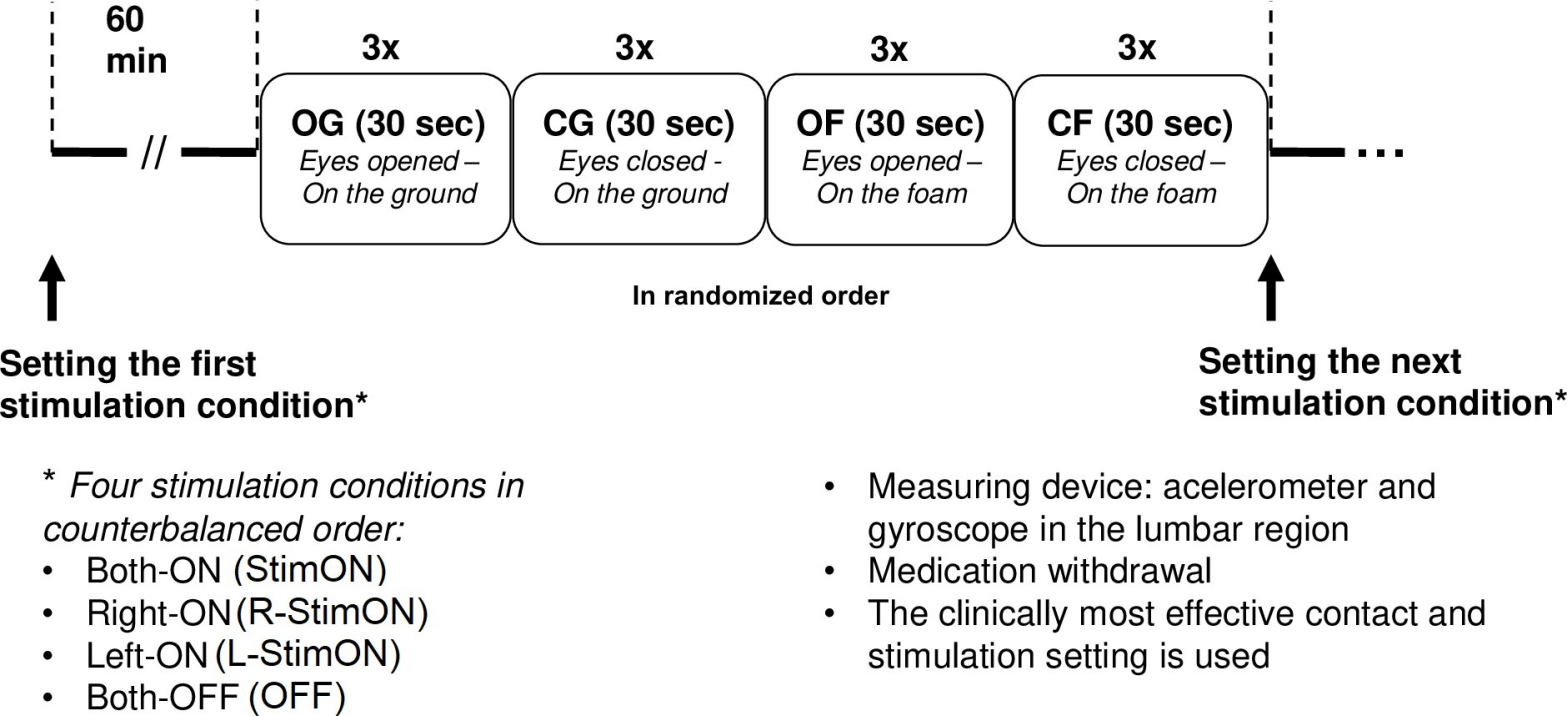
Measurement protocol.

The patients were on not less than a 12h long medication withdrawal at the measurement. We screened four stimulation conditions sequentially, in counterbalanced order: bilateral stimulation OFF (OFF), bilateral stimulation ON (StimON), unilateral right-sided (R-StimON), and left-sided (L-StimON) stimulation ON. We stimulated the clinically used contacts during the complete study, with the stimulation parameters used for therapeutic purposes. A 1-hour time interval was maintained as a washout period between testing in two-different stimulation conditions. Patients and controls repeated each ICTSIB test three times one after another and the average values of the three trials were further analyzed to increase reliability. Study protocol is presented in Fig 1.

### The outcome measure and the collected clinical factors

We calculated sway (m^2^/s^4^; the area of the 95% confidence ellipse, average of the three trials) in each task and combined sway (mean of sway values measured in the four ICTSIB tasks) values in StimON, OFF, R-StimON and L-StimON stimulation conditions with Mobility Lab Software (APDM Inc.). We have chosen this parameter because it characterizes the resulting degree of imbalance in both anterior-posterior and medial-lateral directions [17]. This parameter was shown to have good test-retest reliablility in both the PD and control group, and correlated well with the postural instability and gait disability subscore of the Unified Parkinson’s Disease Rating Scale (UPDRS) scale [35]. It had been earlier validated against the center of pressure displacement measured by a force plate [35].

We determined the International Parkinson and Movement Disorders Society (MDS)-UPDRS III. scores in StimON and OFF stimulation conditions at the time of measurement. We collected the following disease-related parameters: age, disease duration, Hoehn-Yahr stage before and one year after operation, time elapsed since operation, the levodopa responsiveness calculated from the rate of UPDRS III scores in preoperative MED ON and OFF state (dopamine agonists were only stopped one day before the test because patients did not tolerate the discomfort). We determined the stimulation responsiveness from the ratio of the postoperative MDS-UPDRS III. scores in MED OFF-StimON and MED OFF-STIM OFF states, at the time of measurement.

### Anatomical localization of the active DBS contact

We specified the anatomical location of the active contacts. The postoperative CT scans acquired at least 3 months after lead implantation were co-registered with anatomical T1 images using the following parameters: FMRIB Software Library ver. 5.0.9., FLIRT toolbox, linear registration, 6 degrees of freedom, mutual information. Coordinates of the active contacts were calculated using Euclidean vectorial calculations by selecting the most distal and proximal points along the lead. The contact distances were determined according to physical characteristics of the lead (Medtronic Minneapolis, 3389–28, 1.5 mm contact length, 0.5 mm interconnect interspace). The reference point in the dorsolateral STN has been identified as a mathematical center point of the motor portion according to the Atlas [36]. MNI2009 T1 images were co-registered to T1 anatomical images. Mathematical center points of the motor STN regions were warped to an anatomical T1 space using the warp field obtained during non-linear registration (FNIRT, FMRIB Software Library ver. 5.0.9). Distances between the active contacts and the warped motor centers were calculated in each plane of the three-dimensions in millimeters.

### Statistical comparisons

We performed statistical analysis (Table 1) with the Tibco Statistica software (version: 13.5.0.17) on the sway values and the collected clinical data (Tables 2 and S2).

**Table 1:** Summary of the performed statistical tests.

Analyzed variables	Statistical test	Auxiliary test
All data set, distribution fitting	Kolmogorov-Smirnov test	
Sway and combined sway values PD-Control	One-tailed Student t-test	Bonferroni correction
Sway in the tasks and stimulation conditions within PD and control group separately	ANOVA for repeated measures	Neumann-Keuls post hoc test
StimON/OFF combined sway ratio, clinical parameters: worsening-improving PD group	Mann-Whitney U test	
StimON/OFF sway ratio in the 4 tasks within the improving and worsening subgroup separately	Wilcoxon signed-rank test	Bonferroni correction
StimON/OFF sway ratio in the 4 tasks improving-worsening subgroup	Mann-Whitney U test	Bonferroni correction
Age-combined sway in StimON, OFF and controls	Pearson correlation test	
Combination of clinical data predicting combined sway	Cluster analysis Support Vector Regression (SVR) analysis	

**Table 2:** Demographics and clinical data of the recruited patients.

Feature		Values; median and (IQR)
Disease duration		13 (11–18) years
Time since surgery		26 (14.5–43) months
Levodopa equivalent dose	Preoperative	915 (617–1175) mg
	At the study	266 (200–450) mg
Preoperative UPDRS III. score	MED-OFF	28 (23–50) points
	MED-ON	6 (1.5–12.5) points
MDS-UPDRS III. score at the study	MED-OFF, Stim-OFF	33 (22.5–45) points
	MED-OFF, Stim-ON	13 (7–17) points
Hoehn-Yahr stage	Preoperative	3 (2.5–3)
	One year after operation	1 (1–1.5)
Parkinson’s Disease Questionnaire (*PDQ-39) single index score*	Preoperative	25.4 (14.5–33.9)
	One year after operation	20.05 (11.5–25.1)

^**UPDRS: Unified Parkinson’s Disease Rating Scale**^

Normal distribution of the data was first determined with the Kolmogorov-Smirnov test; according to the results, we used parametric or non-parametric statistical tests. We did not exclude outlier values.

The combined sway values and the sway values in the tasks, in the different stimulation conditions were compared with control values using the one-tailed unpaired Student t-test. The p value was determined after a Bonferroni correction. Sway values in the tasks and the stimulation conditions were compared with ANOVA for repeated measures within the PD and control group separately. The within factors were as follows: TASK in both groups with an added STIMULATION CONDITION in the PD group. For multiple comparisons we used the Newman-Keuls test.

We divided the patient group into two subgroups as follows: improving balance (StimON/OFF ratio ≤ 1) and worsening balance (StimON/OFF ratio > 1) after switching the stimulation on. We used the Mann-Whitney U test to compare StimON/OFF ratio, age, disease duration, preoperative and postoperative Hoehn-Yahr stages, time elapsed after operation, the preoperative UPDRS III MED-OFF scores, the preoperative levodopa responsiveness, the stimulation responsiveness and the distances of the active contact from the center point of dorsolateral STN in the three dimensions in the two subgroups. StimON/OFF sway ratio was compared between the sensory tasks within subgroups with Wilcoxon signed-rank test and between subgroups with Mann-Whitney U test. The level of significance was adjusted with Bonferroni correction.

We calculated a correlation between age and combined sway values with the Pearson Correlation test in the controls and patients in StimON and OFF stimulation conditions.

The level of significance was set to p = 0.05.

### The Support Vector Regression (SVR) analyses

We used the Support Vector Regression (SVR) analysis to assess relationship between combined sway and the clinical variables because their association was nonlinear. We aimed to find combinations of two variables as best predictors of imbalance. To evaluate the incremental diagnostic value of pairs of parameters, we performed a 2-step procedure. First, we performed a cluster analysis by grouping all possible combinations (always a pair) of variables to identify pairs with an area under the curve (AUC) > 0.5. Cumulative sums were estimated between 2 parameters by normalizing each parameter to the mean value before summation. Cumulative sums were built for the combination of disease duration and the differences in the Euclidean distance separately for each direction. Second, we built the composite score by estimating the error for those combinations that had survived the first step, and by assigning the weights based on the least error. The composite score represents a four-predictor combination model that includes disease duration and all the distances in the three directions X, Y and Z. The same procedure was followed for the preoperative levodopa responsiveness and the stimulation responsiveness (%).

The Support Vector Regression (SVR) analysis–representing a machine‐learning‐based multiple regression method–could associate the observed and trained values and present the regression coefficient for the accuracy of the prediction [37]. In this study, a data-driven regression model was implemented without explicitly stating a functional form indicating a nonparametric technique.

In short, the algorithm looks for an optimally separating threshold between the two data sets by maximizing the margin between the classes’ closest points. The points lying on the boundaries are called support vectors and the middle of the margin is the optimal separating threshold. Since, in most cases, using a linear separator is not ideal, a projection into a higher dimensional space was performed, whereby the data points effectively become linearly interrelated. Here, we have used the radial basis function kernel for this projection due to its good performance as discussed in [38] and the grid search (min = 1; max = 10) to find the few optimal input parameters namely R (type of regression algorithm; 1 to 1000) and gamma (0.25). The selection was checked by a 10-fold cross validation by taking 75% of the data for training and 25% for testing. A soft-margin classifier of the calculated independent variables was used for every parameter, and spurious correlations were weighted by a penalty constant P. In order to optimize regression accuracy, this was calculated for every regressor. The validation scheme was used to assess whether the included independent parameters that were retinal layers survived in the linear regression. Additionally, we used two parameters as confounders in the analyses, namely, age and tremor.

## Results

### Clinical data

The characteristics of the patient group are summarized in Table 2. There were 5 females and 19 males in the PD group, and 13 females and 11 males in the control group. Parameters of the stimulation are summarized in S2 Table.

### Sway in the four tasks, combined sway

**Fig 2:**
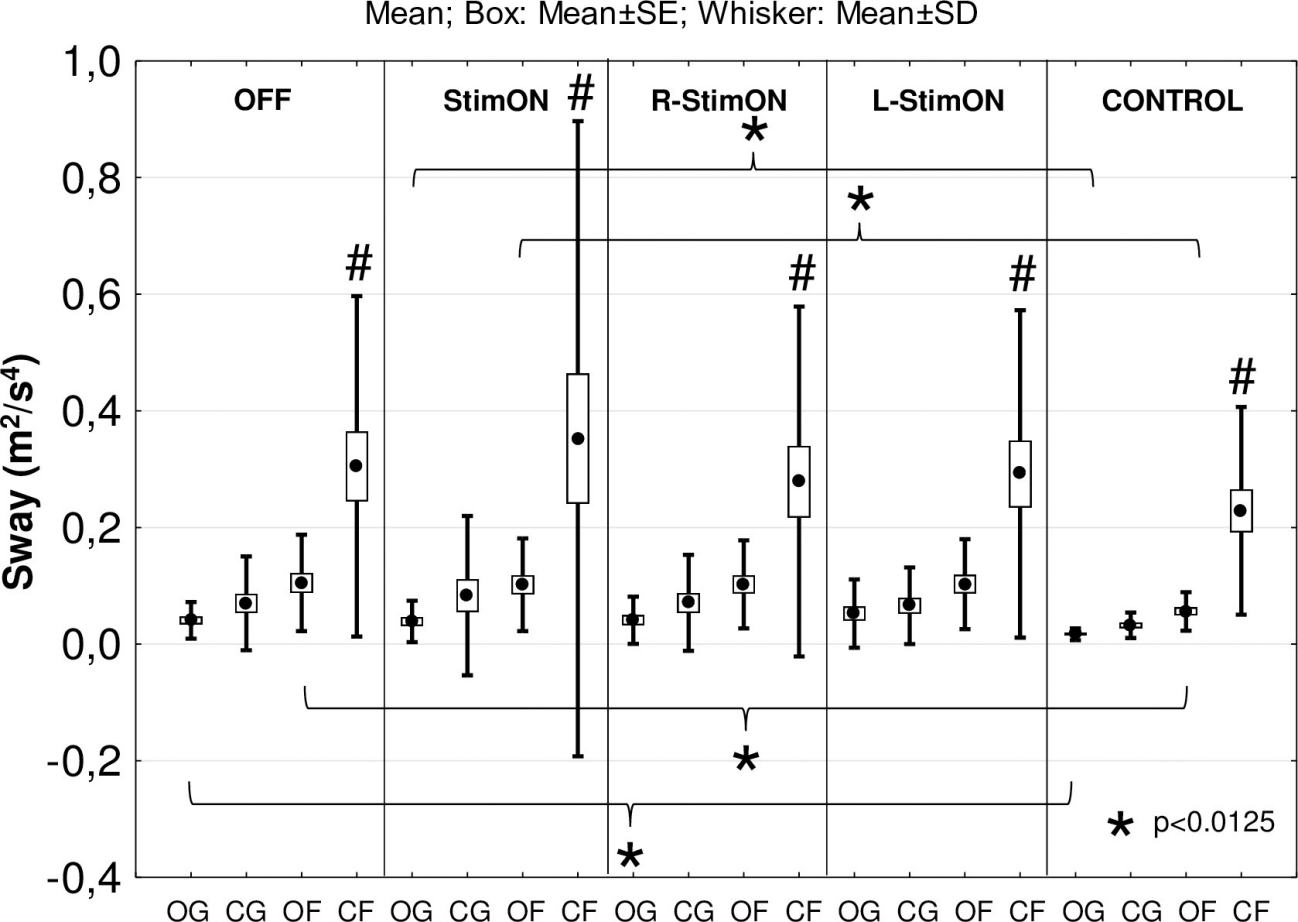
Sway values in the four stimulation conditions and the four tasks. Within the patient and the control group, the sway was significantly larger in the closed eyes-foam task (signed with #) than in the other tasks. The sway in the eyes open-ground and eyes open-foam task was higher in the patient group in OFF and StimON stimulation conditions than in the control group. OG: Eyes open-ground; CG: Eyes closed-ground; OF: Eyes open-foam; CF: Eyes closed-foam.

Sway values were significantly higher in the eyes closed-foam task than in the other tasks in the PD (TASK within factor: F_3-69_ = 14.54; p<0.01 in all comparisons) and the control group (F_3-69_ = 30.82; p<0.01 in all comparisons). Sway in the eyes open-ground and eyes open-foam task was significantly larger in the PD group in OFF (p<0.001 and p = 0.01 sequentially) and StimON (p = 0.006 and p = 0.012 sequentially) stimulation conditions than in controls (corrected p<0.0125; Fig 2). Stimulation conditions did not significantly influence the sway values on the PD group level (STIMULATION within factor: F_3-69_ = 0.396; p = 0.76).

**Fig 3:**
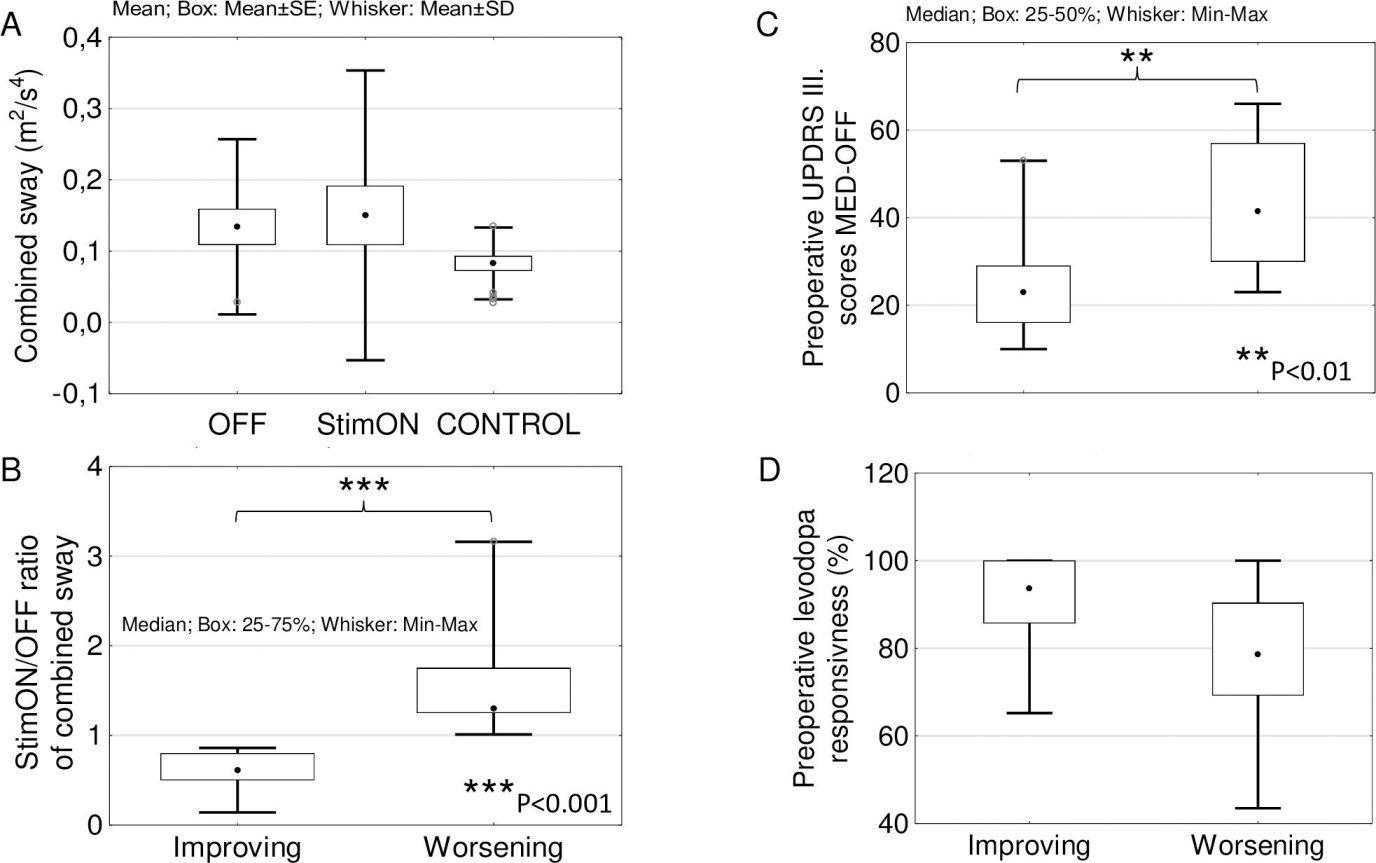
Correspondence between combined sway, symptom severity and levodopa responsiveness. (A) Combined sway on the group level did not differ in the StimON and OFF condition in the patient group and the controls. (B) Stimulation-induced improving and worsening balance subgroups was created based on the StimON/OFF ratio of the combined sway values. (C) UPDRS III scores in medication OFF state was significantly higher representing more severe motor symptoms in the worsening compared to the improving subgroup. (D) Levodopa responsiveness did not distinguish the two subgroups; however, it was higher than 60% in the improving subgroup and higher than 40% in the worsening subgroup.

Combined sway values (average of the sway in the OG, OF, CG and CF tasks) did not differ in PD in the OFF and StimON state and in controls (p>0.04 in all comparisons; level of significance: p = 0.005; Fig 3A) on the group level.

### Comparison of subgroups with improving and worsening balance due to stimulation

**Fig 4:**
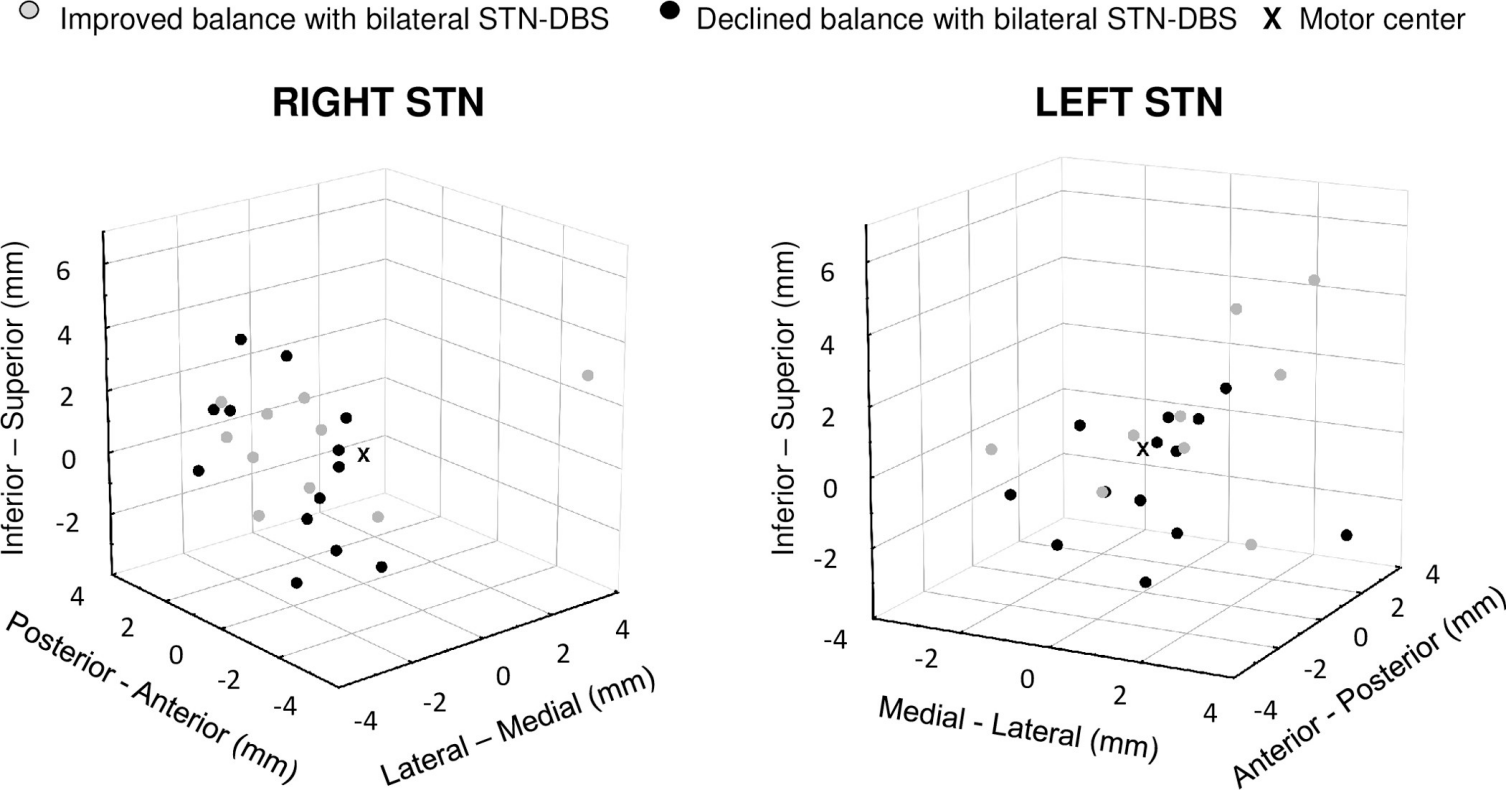
Active contact locations plotted relative to the center point of the dorsolateral STN. The active contact locations (median values in the right STN: x: 0.58mm, y: -1.81mm, z: 0.61mm; left STN: x: 0.57mm, y: -1.05mm, z: 0.67mm) are presented; where x axis represents medial-lateral, the y axis the anterior-posterior, and the z axis the superior-inferior plane. Locations are presented in grey colour if they belong to patients with improving balance when switching bilateral STN DBS on, and in black in case of bilateral STN DBS-induced imbalance. The center point of the dorsolateral STN as the point of reference is indicated with an asterisk in the coordinate system.

**Fig 5:**
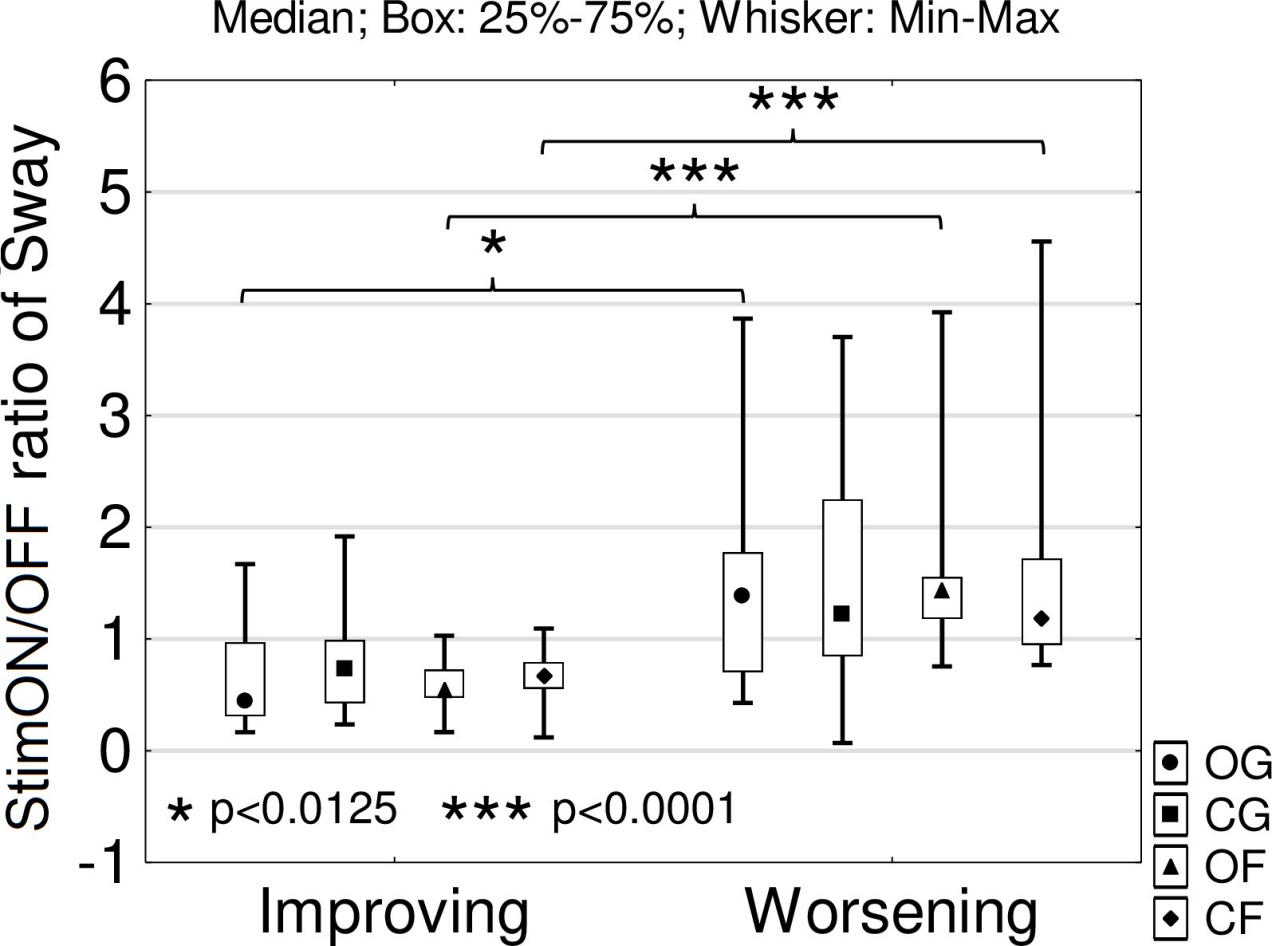
StimON/OFF sway ratio in the sensory conflict situations in the stimulation-induced improving and worsening subgroup. The subgroup with worsening balance had larger stimulation-induced instability in the tests except the eyes-closed, ground trials. The group with worsening balance especially could not compensate the dynamic changes of proprioceptive feedback processes. OG: Eyes-opened, ground; CG: Eyes-closed, ground; OF: Eyes-opened, foam; CF: Eyes-closed, foam.

To explore individual balance changes due to the stimulation, we created two subgroups of PD patients according to the StimON/OFF sway ratio differences as follows: improving (n = 10) and worsening subgroup (n = 12; sway ratio difference: p<0.001; Fig 3B). Neither did age, disease duration, time since surgery, the preoperative levodopa responsiveness, nor did stimulation responsiveness differ significantly in the two subgroups (Table 3). The Hoehn-Yahr stage at the preoperative phase was more advanced in the worsening than in the improving group (Table 3). Active contact distances from the center point of dorsal STN were significantly different only along the z axis in the left STN in the two subgroups, which suggested that there was more inferior stimulation in the left STN in the worsening group (Table 3, Fig 4). We analyzed the effect of bilateral STN DBS (StimON/OFF sway ratio) on sway in the four tasks. The StimON/OFF sway ratio was similar in the four tests in the subgroups; however, it was larger in the worsening compared to the improving subgroup, especially during dynamic changes of proprioceptive sensory feedback and suspended visual information (stance on the foam with closed eyes; Fig 5).

**Table 3:** Comparison of factors in the patients’ subgroups with improving and worsening balance with stimulation ON.

Feature	median (IQR) improving group	median (IQR) worsening group	Mann-Whitney U test, p value
Age (years)	62.5(59–69)	65(63–69)	0.56
Disease duration (years)	13(11–18)	13.5(11–18)	0.88
Hoehn-Yahr stage preoperative	2.5(2.5–3)	3(3–3.5)	***0*.*01***
Hoehn-Yahr stage one year after operation	1(1–1.5)	1(1–1.5)	0.67
Time since surgery (months)	25.5(16–55)	14.5(12–40)	0.09
Stimulation response at the study (%)	66.5(62.7–83.3)	60(42.2–68)	0.08
Location distance from center of dorsal STN, right side (mm)			
x	0.47(-0.9–0.63)	1.31(-0.06–2.26)	0.23
y	-1.86(-2.52- -1.06)	-1.75(-2.42- -0.22)	0.79
z	1.41(-0.75–1.61)	0.19(-1.37–1.37)	0.18
Location distance from center of dorsal STN, left side (mm)			
x	1.19(0.35–2.75)	0.44(-0.42–1.99)	0.23
y	-1.05(-2.43- -0.58)	-1.04(-2.04–0.56)	0.58
z	1.07(0.78–3)	-0.55(-1.64–1.01)	***0*.*04***

_**x: Medial-lateral, y: Anterior-posterior, z: Superior-inferior (Fig**_ _**4**__**).**_

### Prediction analysis

**Fig 6:**
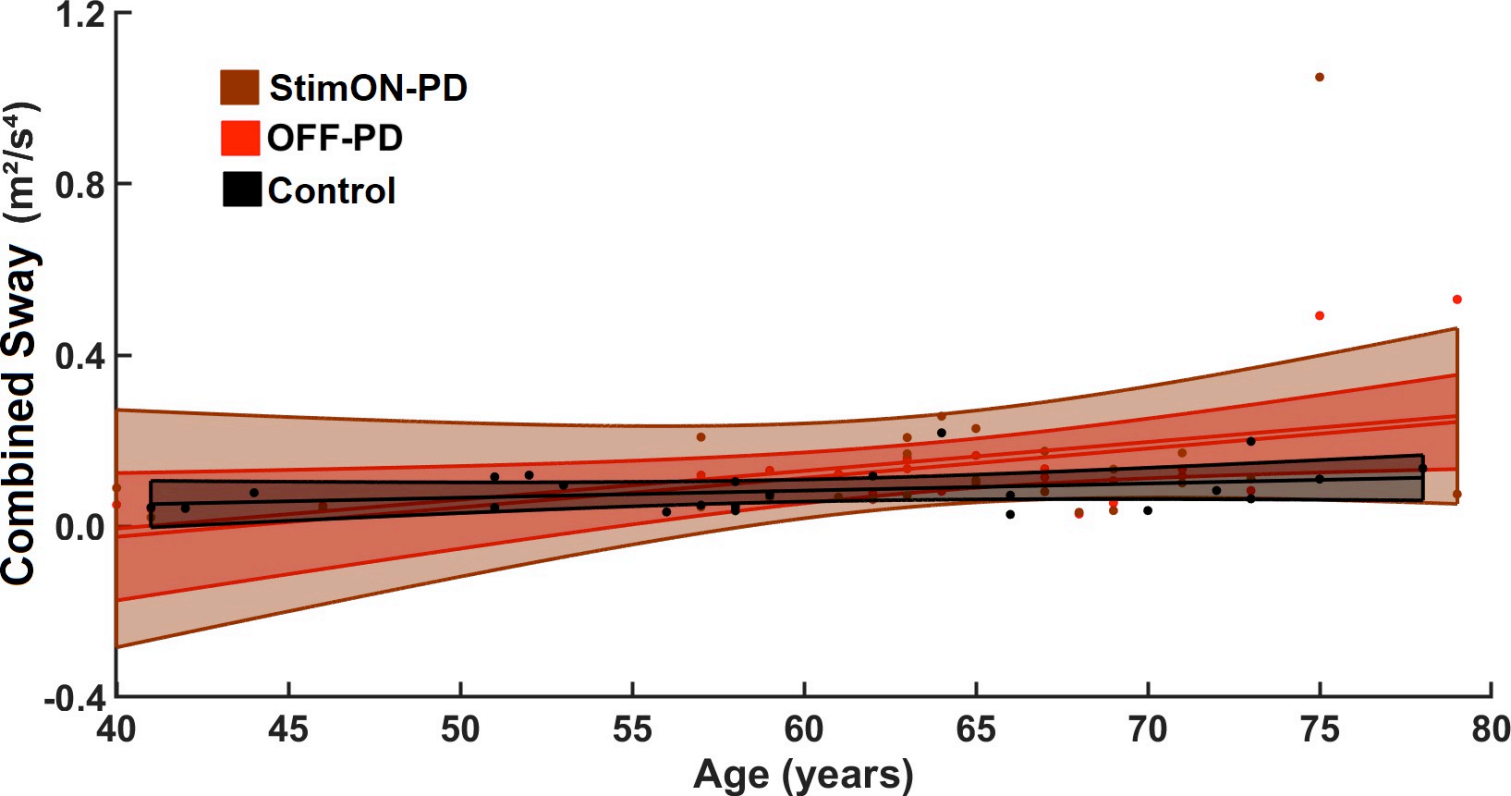
Age-related changes in combined sway values. The linear regressions between the age and the combined sway values are presented in the Parkinson group during bilateral stimulation ON (StimON)-PD, no stimulation (OFF)-PD, and in the healthy control group (Control). Combined sway worsened with age in the PD group in the StimON and OFF stimulation conditions, but not in the control group.

Before prediction analysis, we explored the relations between age and combined sway values (Fig 6). They correlated in the PD group but not in the healthy control group (StimON-PD: r = 0.312, p = 0.023; OFF-PD: r = 0.246, p = 0.043; HC: r = 0.156, p = 0.465). To exclude the effect of age from the prediction analysis, we used age as a confounder.

### SVR results

First, we analyzed the predictive power for the composite score, which was comprised of the disease duration and the difference in Euclidean Vectorial Distance (ED) (X, Y and Z), to predict R-StimON/OFF combined sway. We found a regression coefficient of 0.65 (p < 0.001), which indicated a higher R-StimON/OFF combined sway associated with longer disease duration and higher ED. In the support vector mashines approach, disease duration and ED were 85% accurate at predicting the R-StimON/OFF combined sway values. We also found significant association between the disease duration and ED, for predicting the L-StimON/OFF combined sway with a regression coefficient of 0.58 (p < 0.001) and a 78% predictive accuracy rate. This pair did not predict StimON/OFF sway significantly, the regression coefficient was only 0.48 (p = 0.23).

**Fig 7:**
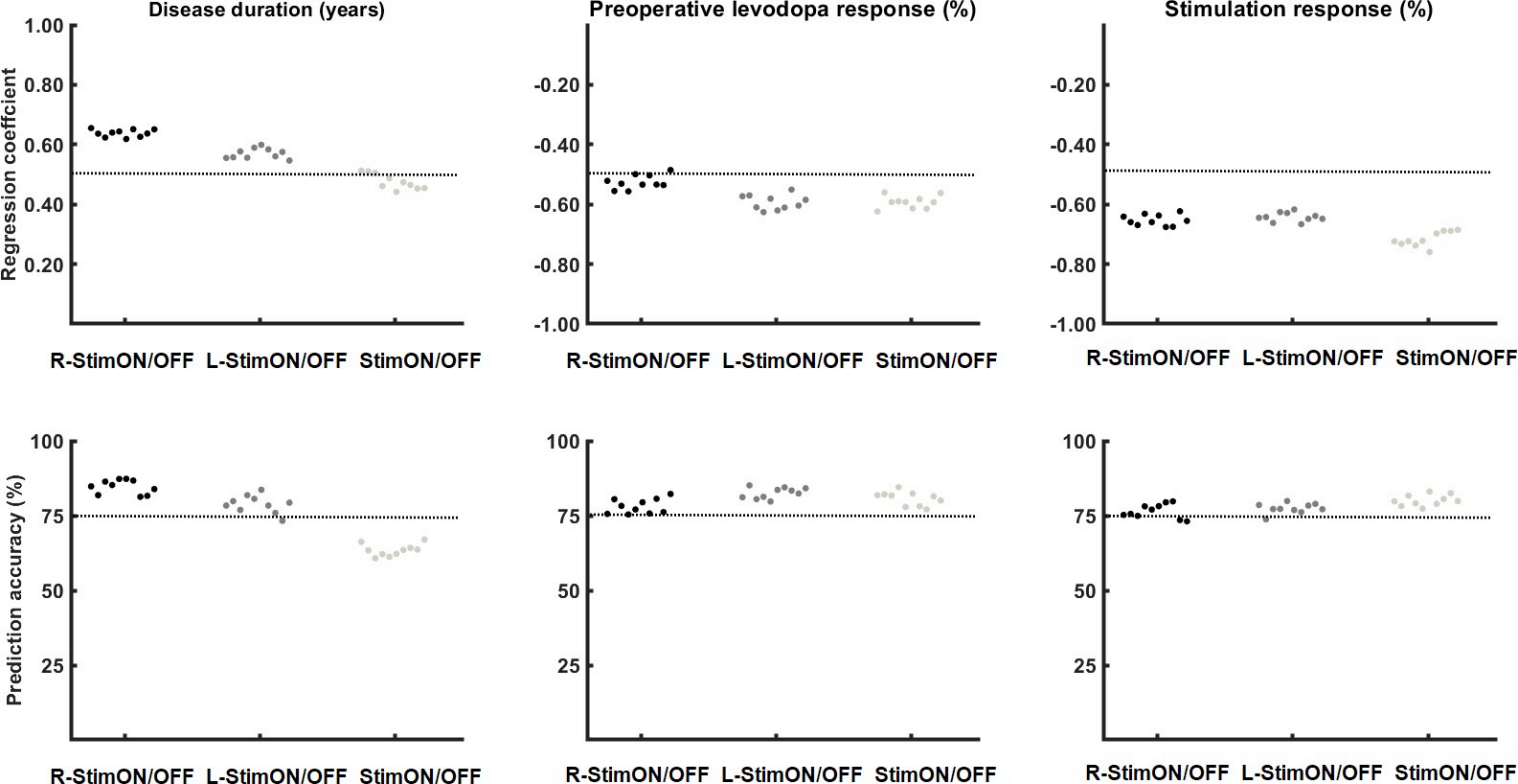
Prediction coefficients and the accuracy of disease duration, the levodopa and stimulation responsiveness. The 10-fold cross validated SVR results are shown separately for validation of each factor predicting the effect of the right (R-StimON), left (L-StimON), and both sided (StimON) stimulation on balance.

Second, we analyzed the predictive power for the composite score, which comprised of the preoperative levodopa responsiveness and the difference in ED (X, Y and Z) to predict the L-StimON/OFF combined sway. It showed the best prediction regression coefficient of -0.61 (p < 0.001), which indicated that higher L-StimON/OFF combined sway was associated with lower preoperative levodopa responsiveness. This pair predicted the StimON/OFF combined sway value with a regression coefficient of -0.72 (p<0.001) and an 82% predictive accuracy. To verify and validate the SVR results, we show the 10-fold cross validation results for each parameter in Fig 7, Table 4.

**Table 4:** The area under the curve (AUC) from the first step for each combination are listed separately for each independent variable namely disease duration (DD), preoperative levodopa responses (PLR) and stimulation response (SR).

Disease Duration (DD)		Preoperative levodopa responses (PLR)		Stimulation responses (SR)	
Combination	AUC	Combination	AUC	Combination	AUC
X+Y	0.46	X+Y	0.42	X+Y	0.37
Y+Z	0.42	Y+Z	0.38	Y+Z	0.38
X+Z	0.45	X+Z	0.36	X+Z	0.29
X+DD	0.47	X+PLR	0.38	X+SR	0.39
Y+DD	0.49	Y+PLR	0.41	Y+SR	0.42
Z+DD	0.42	Z+PLR	0.43	Z+SR	0.43
ED+DD	0.56	ED+PLR	0.54	ED+SR	0.58

## Discussion

Our results confirm that the long-term effects of STN DBS on quiet stance balance are multifactorial in Parkinson’s disease. The combination of the disease duration, the preoperative levodopa responsiveness, and the stimulation responsiveness predict quiet stance postural instability the best during long-term bilateral subthalamic stimulation. On the individual level, a worsening of quiet stance balance while switching the stimulation on can be associated with more severe symptoms of the disease; this mechanism may be primarily due to the attenuated dependency on the proprioceptive and additionally the visual sensory feedback information.

### Quiet stance in the tasks and stimulation conditions

On the PD group level, sway did not differ in the stimulation conditions, i.e., OFF, StimON, R-StimON, and L-StimON. We noticed that sway worsens or improves after switching on the stimulation in different patients and that group comparisons obscure individual stimulation effects. Therefore, we devided patients into two groups in which combined sway worsened or improved with stimulation in the StimON compared to the OFF condition. A higher Hoehn-Yahr stage and preoperative UPDRS III scores in medication off phase confirmed a more severe disease in the worsening than in the improving group, suggesting that disease progression is the major cause of stimulation-induced imbalance. In the two subgroups, the worsening or improving effect of stimulation was only partial in unilateral stimulation as compared with the effect of bilateral stimulation.

We explored combination of clinical factors that are predictive for stimulation-induced imbalance observed on the individual level.

### The effect of disease duration and the severity of motor symptoms

Disease duration combined with the levodopa and the stimulation responsiveness was predictive for imbalance during a long-term STN DBS in our study. These results are in line with other results that express that disease duration is a potential predictor of non-levodopa-responsive axial motor impairment in PD independent from the effect of age [39]. As aging is an additional aggravating factor of postural instability [13,40], we set it as a confounder in the prediction analysis; therefore, we excluded its effects when estimating the consequences of STN DBS.

### The effect of levodopa responsiveness

We show that better preoperative levodopa responsiveness is less likely coupled with stimulation-induced imbalance after surgery. The clinical guideline of indications for STN DBS surgery [32] suggests at least a 33% improvement of the UPDRS III motor scores during a preoperative levodopa challenge test [41], although a 50% improvement has been associated with a better postoperative outcome [42,43]. Comparing the subgroups that improved and worsened when stimulation was turned on, the minimum levodopa responsiveness in the first group was 65%, while in the second group it was 43%. This confirms that some components of balance regulation are related to dopa-responsive and, in turn, stimulation responsive motor symptoms [1,20].

### The determinants of the stimulation responsiveness

Stimulation responsiveness is closely related to the levodopa responsiveness [44], the stimulating contact location, and the programming approach [45].

The relationship between the site of stimulation and the change in balance is not unequivocal. A better overall motor improvement in the UPDRS III. scores were documented in the long-term when the active contact located in the dorsolateral STN [18]. Stimulation in the dorsal part was more effective than in the ventral part of the STN for dynamic balance in a group of 10 PD patients [46]. In contrast, another study described a similar dynamic balance irrespective of dorsal or ventral simulation while testing 23 PD patients [25]. In our study, the median absolute distance between the active contact and the center of dorsolateral motor STN was no more than 1.9mm on the right and 1.2mm on the left side, which had no significant role in predicting stimulation-induced imbalance. However, in the worsening subgroup, location of the stimulation was significantly more ventral in the left STN than what was calculated in the improving group, which is in line with the results mentioned above. It was already speculated that a more ventral stimulation might affect PPN projections provoking imbalance [47].

Assessing the programming approach, it has been observed that bilateral stimulation has a better effect on postural stability than unilateral stimulation [22] and that significant asymmetry in the stimulations voltage between the two sides may worsen the interlimb coordination [48]. The patients in this study were bilaterally stimulated, with the median interhemispheric difference in the stimulation voltage being 0 (IQR: 0–0.01); therefore, we can confirm that the programming strategy did not influence the results.

### Sensory deficits worsen stimulation-induced instability

We employed kinematic analysis of quiet stance in different sensory conflict situations to characterize balance strategies better [35]. In earlier studies, an increase has been reported in visual dependency to maintain balance in PD [10,13], which worsens after STN stimulation [1]. Our study showed that sway increases, especially while standing on foam in patients with stimulation induced worsening of balance, which suggests an increased dependency on proprioceptive information. However, we also confirmed a stimulation-induced increase in visual dependency. These results highlight that more attention should be paid to associated diseases during the preoperative clinical evaluation, such as polyneuropathy, which may further exacerbate imbalance.

### Limitations

A limitation of our study was that we only analyzed the quiet stance in sensory conflict situations when exploring the effect of subthalamic stimulation. However, other balance elements, such as dynamic balance, may conteract and should also be calculated in other studies. Another limitation is the number of subjects, testing of higher number of patients would be beneficial.

In conclusion, we showed that the effect of STN DBS on balance is individually variable. Younger age, less severe motor symptoms, and preferably, more than 60% improvement in UPDRS III scores during the levodopa challenge test are predictive for less balance problems evoked by the bilateral STN DBS. We show that dependency on the proprioceptive information rises with stimulation, besides visual dependency. This should be considered during the preoperative clinical evaluation of the patients. And as the disease progresses during chronic therapy, the testing of specific programming strategies becomes necessary.

## Supporting information

S1 Table Studies on the different balance components used various assessment methods to investigate the effect of subthalamic stimulation. (DOCX)

S2 Table The analyzed sway value and clinical parameters. (XLSX)

## References

[1] Schoneburg B, Mancini M, Horak F, Nutt JG. Framework for understanding balance dysfunction in Parkinson’s disease. Mov Disord. 2013;28(11):1474–82. Epub 2013/08/09. doi: 10.1002/mds.25613 ; PubMed Central PMCID: PMC4001822. 23925954 PMC4001822

[2] Schrag A, Horsfall L, Walters K, Noyce A, Petersen I. Prediagnostic presentations of Parkinson’s disease in primary care: a case-control study. Lancet Neurol. 2015;14(1):57–64. Epub 2014/12/02. doi: 10.1016/S1474-4422(14)70287-X . 25435387

[3] Halmi Z, Dinya E, Málly J. Destroyed non-dopaminergic pathways in the early stage of Parkinson’s disease assessed by posturography. Brain Res Bull. 2019;152:45–51. Epub 2019/07/12. doi: 10.1016/j.brainresbull.2019.07.001 . 31295517

[4] Frenklach A, Louie S, Koop MM, Bronte-Stewart H. Excessive postural sway and the risk of falls at different stages of Parkinson’s disease. Mov Disord. 2009;24(3):377–85. Epub 2008/10/31. doi: 10.1002/mds.22358 . 18972546

[5] Bloem BR, Beckley DJ, van Dijk JG, Zwinderman AH, Remler MP, Roos RA. Influence of dopaminergic medication on automatic postural responses and balance impairment in Parkinson’s disease. Mov Disord. 1996;11(5):509–21. Epub 1996/09/01. doi: 10.1002/mds.870110506 . 8866492

[6] Bohnen NI, Müller ML, Koeppe RA, Studenski SA, Kilbourn MA, Frey KA, et al. History of falls in Parkinson disease is associated with reduced cholinergic activity. Neurology. 2009;73(20):1670–6. Epub 2009/11/18. doi: 10.1212/WNL.0b013e3181c1ded6 ; PubMed Central PMCID: PMC2788804. 19917989 PMC2788804

[7] Shulman LM, Gruber-Baldini AL, Anderson KE, Vaughan CG, Reich SG, Fishman PS, et al. The evolution of disability in Parkinson disease. Mov Disord. 2008;23(6):790–6. Epub 2008/03/26. doi: 10.1002/mds.21879 . 18361474

[8] Johnson L, James I, Rodrigues J, Stell R, Thickbroom G, Mastaglia F. Clinical and posturographic correlates of falling in Parkinson’s disease. Mov Disord. 2013;28(9):1250–6. Epub 2013/04/24. doi: 10.1002/mds.25449 . 23609352

[9] Venhovens J, Meulstee J, Bloem BR, Verhagen WI. Neurovestibular analysis and falls in Parkinson’s disease and atypical parkinsonism. Eur J Neurosci. 2016;43(12):1636–46. Epub 2016/04/12. doi: 10.1111/ejn.13253 . 27062368

[10] Rinalduzzi S, Trompetto C, Marinelli L, Alibardi A, Missori P, Fattapposta F, et al. Balance Dysfunction in Parkinson’s Disease. BioMed Research International. 2015;2015:434683. doi: 10.1155/2015/434683 25654100 PMC4310258

[11] Camicioli R, Majumdar SR. Relationship between mild cognitive impairment and falls in older people with and without Parkinson’s disease: 1-Year Prospective Cohort Study. Gait Posture. 2010;32(1):87–91. Epub 2010/05/04. doi: 10.1016/j.gaitpost.2010.03.013 . 20434917

[12] Kammermeier S, Maierbeck K, Dietrich L, Plate A, Lorenzl S, Singh A, et al. Qualitative postural control differences in Idiopathic Parkinson’s Disease vs. Progressive Supranuclear Palsy with dynamic-on-static platform tilt. Clin Neurophysiol. 2018;129(6):1137–47. Epub 2018/04/10. doi: 10.1016/j.clinph.2018.03.002 . 29631169

[13] Ickenstein GW, Ambach H, Klöditz A, Koch H, Isenmann S, Reichmann H, et al. Static posturography in aging and Parkinson’s disease. Front Aging Neurosci. 2012;4:20. Epub 2012/08/14. doi: 10.3389/fnagi.2012.00020 ; PubMed Central PMCID: PMC3412413. 22888319 PMC3412413

[14] Barbosa AF, Chen J, Freitag F, Valente D, Souza CO, Voos MC, et al. Gait, posture and cognition in Parkinson’s disease. Dement Neuropsychol. 2016;10(4):280–6. Epub 2016/10/01. doi: 10.1590/s1980-5764-2016dn1004005 ; PubMed Central PMCID: PMC5619266. 29213470 PMC5619266

[15] Horak FB, Shupert CL, Mirka A. Components of postural dyscontrol in the elderly: a review. Neurobiol Aging. 1989;10(6):727–38. Epub 1989/11/01. doi: 10.1016/0197-4580(89)90010-9 . 2697808

[16] Rocchi L, Chiari L, Horak FB. Effects of deep brain stimulation and levodopa on postural sway in Parkinson’s disease. J Neurol Neurosurg Psychiatry. 2002;73(3):267–74. Epub 2002/08/20. doi: 10.1136/jnnp.73.3.267 ; PubMed Central PMCID: PMC1738049. 12185157 PMC1738049

[17] Workman CD, Thrasher TA. The influence of dopaminergic medication on balance automaticity in Parkinson’s disease. Gait Posture. 2019;70:98–103. Epub 2019/03/06. doi: 10.1016/j.gaitpost.2019.02.015 . 30836253

[18] Aviles-Olmos I, Kefalopoulou Z, Tripoliti E, Candelario J, Akram H, Martinez-Torres I, et al. Long-term outcome of subthalamic nucleus deep brain stimulation for Parkinson’s disease using an MRI-guided and MRI-verified approach. J Neurol Neurosurg Psychiatry. 2014;85(12):1419–25. Epub 2014/05/03. doi: 10.1136/jnnp-2013-306907 ; PubMed Central PMCID: PMC4451170. 24790212 PMC4451170

[19] Krack P, Batir A, Van Blercom N, Chabardes S, Fraix V, Ardouin C, et al. Five-year follow-up of bilateral stimulation of the subthalamic nucleus in advanced Parkinson’s disease. N Engl J Med. 2003;349(20):1925–34. Epub 2003/11/14. doi: 10.1056/NEJMoa035275 . 14614167

[20] St George RJ, Nutt JG, Burchiel KJ, Horak FB. A meta-regression of the long-term effects of deep brain stimulation on balance and gait in PD. Neurology. 2010;75(14):1292–9. Epub 2010/10/06. doi: 10.1212/WNL.0b013e3181f61329 ; PubMed Central PMCID: PMC3013496. 20921515 PMC3013496

[21] Szlufik S, Kloda M, Friedman A, Potrzebowska I, Gregier K, Mandat T, et al. The Neuromodulatory Impact of Subthalamic Nucleus Deep Brain Stimulation on Gait and Postural Instability in Parkinson’s Disease Patients: A Prospective Case Controlled Study. Front Neurol. 2018;9:906. Epub 2018/11/16. doi: 10.3389/fneur.2018.00906 ; PubMed Central PMCID: PMC6220087. 30429820 PMC6220087

[22] Kumar R, Lozano AM, Sime E, Halket E, Lang AE. Comparative effects of unilateral and bilateral subthalamic nucleus deep brain stimulation. Neurology. 1999;53(3):561–6. Epub 1999/08/17. doi: 10.1212/wnl.53.3.561 . 10449121

[23] De la Casa-Fages B, Alonso-Frech F, Grandas F. Effect of subthalamic nucleus deep brain stimulation on balance in Parkinson’s disease: A static posturographic analysis. Gait Posture. 2017;52:374–80. Epub 2017/01/07. doi: 10.1016/j.gaitpost.2016.12.025 . 28061431

[24] McNeely ME, Earhart GM. Medication and subthalamic nucleus deep brain stimulation similarly improve balance and complex gait in Parkinson disease. Parkinsonism Relat Disord. 2013;19(1):86–91. Epub 2012/08/14. doi: 10.1016/j.parkreldis.2012.07.013 ; PubMed Central PMCID: PMC3508177. 22885253 PMC3508177

[25] McNeely ME, Hershey T, Campbell MC, Tabbal SD, Karimi M, Hartlein JM, et al. Effects of deep brain stimulation of dorsal versus ventral subthalamic nucleus regions on gait and balance in Parkinson’s disease. J Neurol Neurosurg Psychiatry. 2011;82(11):1250–5. Epub 2011/04/12. doi: 10.1136/jnnp.2010.232900 ; PubMed Central PMCID: PMC3250990. 21478202 PMC3250990

[26] Colnat-Coulbois S, Gauchard GC, Maillard L, Barroche G, Vespignani H, Auque J, et al. Bilateral subthalamic nucleus stimulation improves balance control in Parkinson’s disease. J Neurol Neurosurg Psychiatry. 2005;76(6):780–7. Epub 2005/05/18. doi: 10.1136/jnnp.2004.047829 ; PubMed Central PMCID: PMC1739669. 15897498 PMC1739669

[27] Nantel J, McDonald JC, Bronte-Stewart H. Effect of medication and STN-DBS on postural control in subjects with Parkinson’s disease. Parkinsonism Relat Disord. 2012;18(3):285–9. Epub 2011/12/02. doi: 10.1016/j.parkreldis.2011.11.005 . 22130147

[28] Shivitz N, Koop MM, Fahimi J, Heit G, Bronte-Stewart HM. Bilateral subthalamic nucleus deep brain stimulation improves certain aspects of postural control in Parkinson’s disease, whereas medication does not. Mov Disord. 2006;21(8):1088–97. Epub 2006/05/04. doi: 10.1002/mds.20905 . 16671073

[29] St George RJ, Carlson-Kuhta P, Burchiel KJ, Hogarth P, Frank N, Horak FB. The effects of subthalamic and pallidal deep brain stimulation on postural responses in patients with Parkinson disease. J Neurosurg. 2012;116(6):1347–56. Epub 2012/03/20. doi: 10.3171/2012.2.JNS11847 ; PubMed Central PMCID: PMC3465575. 22424564 PMC3465575

[30] St George RJ, Carlson-Kuhta P, Nutt JG, Hogarth P, Burchiel KJ, Horak FB. The effect of deep brain stimulation randomized by site on balance in Parkinson’s disease. Mov Disord. 2014;29(7):949–53. Epub 2014/02/18. doi: 10.1002/mds.25831 ; PubMed Central PMCID: PMC4057940. 24532106 PMC4057940

[31] Johnsen EL. Gait and postural instability in Parkinson’s disease treated with deep brain stimulation of the subthalamic nucleus. Dan Med Bull. 2011;58(10):B4334. Epub 2011/10/07. . 21975157

[32] Defer GL, Widner H, Marié RM, Rémy P, Levivier M. Core assessment program for surgical interventional therapies in Parkinson’s disease (CAPSIT-PD). Mov Disord. 1999;14(4):572–84. Epub 1999/08/06. doi: 10.1002/1531-8257(199907)14:4&lt;572::aid-mds1005&gt;3.0.co;2-c . 10435493

[33] Tamás G, Kelemen A, Radics P, Valálik I, Heldman D, Klivényi P, et al. Effect of subthalamic stimulation on distal and proximal upper limb movements in Parkinson’s disease. Brain Res. 2016;1648(Pt A):438–44. Epub 2016/08/21. doi: 10.1016/j.brainres.2016.08.019 . 27543337

[34] Mancini M, King L, Salarian A, Holmstrom L, McNames J, Horak FB. Mobility Lab to Assess Balance and Gait with Synchronized Body-worn Sensors. J Bioeng Biomed Sci. 2011;Suppl 1:007. Epub 2011/12/12. doi: 10.4172/2155-9538.S1-007 ; PubMed Central PMCID: PMC4062543. 24955286 PMC4062543

[35] Mancini M, Salarian A, Carlson-Kuhta P, Zampieri C, King L, Chiari L, et al. ISway: a sensitive, valid and reliable measure of postural control. J Neuroeng Rehabil. 2012;9:59. Epub 2012/08/24. doi: 10.1186/1743-0003-9-59 ; PubMed Central PMCID: PMC3481400. 22913719 PMC3481400

[36] Accolla EA, Dukart J, Helms G, Weiskopf N, Kherif F, Lutti A, et al. Brain tissue properties differentiate between motor and limbic basal ganglia circuits. Hum Brain Mapp. 2014;35(10):5083–92. Epub 2014/04/30. doi: 10.1002/hbm.22533 ; PubMed Central PMCID: PMC4282398. 24777915 PMC4282398

[37] Drucker H, Burges CJ, Kaufman L, Smola A, Vapnik V. Support vector regression machines. Advances in neural information processing systems. 1997;9:155–61.

[38] Cortes C, Vapnik V. Support-vector networks. Machine learning. 1995;20(3):273–97.

[39] Levy G, Louis ED, Cote L, Perez M, Mejia-Santana H, Andrews H, et al. Contribution of aging to the severity of different motor signs in Parkinson disease. Arch Neurol. 2005;62(3):467–72. Epub 2005/03/16. doi: 10.1001/archneur.62.3.467 . 15767513

[40] Judge JO, King MB, Whipple R, Clive J, Wolfson LI. Dynamic balance in older persons: effects of reduced visual and proprioceptive input. J Gerontol A Biol Sci Med Sci. 1995;50(5):M263–70. Epub 1995/09/01. doi: 10.1093/gerona/50a.5.m263 . 7671028

[41] Saranza G, Lang AE. Levodopa challenge test: indications, protocol, and guide. J Neurol. 2020. Epub 2020/04/26. doi: 10.1007/s00415-020-09810-7 . 32333167

[42] Artusi CA, Lopiano L, Morgante F. Deep Brain Stimulation Selection Criteria for Parkinson’s Disease: Time to Go beyond CAPSIT-PD. J Clin Med. 2020;9(12). Epub 2020/12/10. doi: 10.3390/jcm9123931 ; PubMed Central PMCID: PMC7761824. 33291579 PMC7761824

[43] Deuschl G, Follett KA, Luo P, Rau J, Weaver FM, Paschen S, et al. Comparing two randomized deep brain stimulation trials for Parkinson’s disease. J Neurosurg. 2019;132(5):1376–84. Epub 2019/04/06. doi: 10.3171/2018.12.JNS182042 . 30952118

[44] Charles PD, Van Blercom N, Krack P, Lee SL, Xie J, Besson G, et al. Predictors of effective bilateral subthalamic nucleus stimulation for PD. Neurology. 2002;59(6):932–4. Epub 2002/09/26. doi: 10.1212/wnl.59.6.932 . 12297584

[45] Hartmann CJ, Fliegen S, Groiss SJ, Wojtecki L, Schnitzler A. An update on best practice of deep brain stimulation in Parkinson’s disease. Ther Adv Neurol Disord. 2019;12:1756286419838096. Epub 2019/04/05. doi: 10.1177/1756286419838096 ; PubMed Central PMCID: PMC6440024. 30944587 PMC6440024

[46] Johnsen EL, Sunde N, Mogensen PH, Ostergaard K. MRI verified STN stimulation site—gait improvement and clinical outcome. Eur J Neurol. 2010;17(5):746–53. Epub 2010/03/30. doi: 10.1111/j.1468-1331.2010.02962.x . 20345927

[47] Boonstra TA, van der Kooij H, Munneke M, Bloem BR. Gait disorders and balance disturbances in Parkinson’s disease: clinical update and pathophysiology. Curr Opin Neurol. 2008;21(4):461–71. Epub 2008/07/09. doi: 10.1097/WCO.0b013e328305bdaf . 18607208

[48] Pötter-Nerger M, Volkmann J. Deep brain stimulation for gait and postural symptoms in Parkinson’s disease. Mov Disord. 2013;28(11):1609–15. Epub 2013/10/18. doi: 10.1002/mds.25677 . 24132849

