# Supplementary material for: Factors affecting postural instability after more than one-year bilateral subthalamic stimulation in Parkinson’s disease: A cross-sectional study
Source: PLoS One. 2022 Feb 23;17(2):e0264114. doi: 10.1371/journal.pone.0264114 (PMC8865658; doi:10.1371/journal.pone.0264114)
Supplement: S1 Table — (DOCX) [file pone.0264114.s001.docx]

**S1 Table.** Studies on the different balance components used various assessment methods to investigate the effect of subthalamic stimulation

| **Study** | **No of Patients with DBS target** | **Timing of postoperative assessment after OP** | **Assessment method** | **Assessed**  **parameters** | **Medication, stimulation conditions** | **DBS contact location** | **Stimulation effect on balance** |
| --- | --- | --- | --- | --- | --- | --- | --- |
| St. George et al. 2014^30^ | STN:14  GPI:14  Control PD:9 | <6 months | PIGD items of the UPDRS III. scale  Balance and Gait scales | Scores preoparetive and 6 months after surgery | StimON/MedOFF  StimOFF/MedOFF  StimOFF/MedON  StimON/MedON | No | Levodopa and DBS had positive and additive effects. The effect of Gpi-DBS was preferable more than STN-DBS. |
| St. George et al. 2012^29^ | STN:13  GPI:11  Control PD:9  Control:17 | 6 months | Force plate  EMG  Stance during external pertubations | Automatic postural response stability  Muscle burst duration, latencies, Co-activation index | StimOFF/MedOFF  StimOFF/MedON  StimON/MedON | No | STN-DBS but not GPi-DBS worsened the stability after 6 months. |
| Nantel et al. 2012^27^ | STN:28  PD:101  Control:23 | 9.6±2.4 months | Force plate  Quiet stance | Root mean square, mean velocity of CoP displacement | StimON/MedOFF  StimON/MedON | No | STN-DBS had positive effect. |
| De la Casa Fages et al. 2017^23^ | STN:16  Control:13 | mean:  32 months | Force plate  Quiet stance and dual task | 14 parameters related to CoP sway | StimOFF/MedOFF  StimOFF/MedON  StimON/MedOFF  StimON/MedON | No | STN-DBS had a discrete positive effect. |
| Shivitz et al. 2006^28^ | STN:28 | 6-12 months | PIGD scale  Force plate Stance during external perturbations | CoG angle  CoG sway | StimOFF/MedOFF  StimOFF/MedON  StimON/MedOFF  StimON/MedON | No | STN-DBS improved abnormal sensory aspects of balance. |
| Szlufik et al. 2018^21^ | STN:20 early postoperative  14 late postoperative  Control PD:20 | late postoperative:  median: 30 months | UPDRS-III.  Clinical Balance test  force plate  static and dynamic balance | COP velocity, ellipse area | StimOFF/MedOFF  StimOFF/MedON  StimON/MedOFF  StimON/MedON | No | Positive effect of STN-DBS on static and dynamic balance in first post-operative 9 months period followed by deterioration in StimOFF phase suggesting neuromodulatory effect. |
| McNeely et al. 2011^25^ | STN:23 | >3 months | Mini-Balance Evaluation Systems Test  dynamic balance | scores | Unilateral dorsal StimON  Unilateral ventral StimON  StimOFF | Yes | Dorsal and ventral STN-DBS did not differ significantly. |
| McNeely et a. 2013^24^ | STN:12 | 3 months | Mini-Balance Evaluation Systems Test  dynamic balance | scores | StimON/MedOFF  StimON/MedON  StimOFF/MedOFF  StimOFF/MedON | No | STN-DBS improved the dynamic balance. |
| Colnat-Coulbois et al. 2005^26^ | STN:12 | 6 months | Force plate  Static and dynamic balance | CoP displacement  sway path and area | PreOP MedON  StimON/MedON | No | STN-DBS in combination with l-dopa treatment reduces postural instability. |
| Rocchi et al. 2002^16^ | STN:3  GPi:3  Control:11 | 6 months | Force plate | Variables of CoP displacement | StimON/MedOFF  StimON/MedON  StimOFF/MedOFF  StimOFF/MedON | No | STN- and GPi-DBS improved postural sway. |

_PIGD: postural instability and gait difficulties; CoP: Center of Pressure_
